# HPV types and cofactors causing cervical cancer in Peru

**DOI:** 10.1054/bjoc.2001.1948

**Published:** 2001-09

**Authors:** C Santos, N Muñoz, S Klug, M Almonte, I Guerrero, M Alvarez, C Velarde, O Galdos, M Castillo, J Walboomers, C Meijer, E Caceres

**Affiliations:** Instituto de Enfermedades Neoplásicas ‘Dr Eduardo Cáceres Graziani', Av. Angamos Este 2520, Lima, 34, Peru; International Agency for Research on Cancer, 150 Cours Albert Thomas, Lyon cédex 08, F-69372, France; IARC, University of Bielefeld, PO Box 100131, Bielefeld, D-33501, Germany; Maes-Heller Cancer Research Centre, Av. Angamos Este 2520, Lima, 34, Peru; María Auxiliadora' General Hospital, Lima, Peru; Free University Hospital, Postbus 7057, Amsterdam, MB, NL-1007, The Netherlands

**Keywords:** cervical cancer, human papillomavirus, Peru, case-control

## Abstract

We conducted a hospital-based case-control study in Peru of 198 women with histologically confirmed cervical cancer (173 squamous cell carcinomas and 25 cases of adenocarcinoma/adenosquamous carcinoma) and 196 control women. Information on risk factors was obtained by personal interview. Using PCR-based assays on exfoliated cervical cells and biopsy specimens, HPV DNA was detected in 95.3% of women with squamous cell carcinoma and in 92.0% of women with adenocarcinoma/adenosquamous carcinoma compared with 17.7% in control women. The age-adjusted odds ratio was 116.0 (95% Cl = 48.6–276.0) for squamous cell carcinoma and 51.4 (95% Cl = 11.4–232.0) for adenocarcinoma/adenosquamous carcinoma. The commonest types in women with cervical cancer were HPV 16, 18, 31, 52 and 35. The association with the various HPV types was equally strong for the two most common types (HPV 16 and 18) as for the other less common types. In addition to HPV, long-term use of oral contraceptives and smoking were associated with an increased risk. HPV is the main cause of both squamous cell carcinoma and adenocarcinoma in Peruvian women. © 2001 Cancer Research Campaignhttp://www.bjcancer.com


*British Journal of Cancer* (2001) **85**(7), 966–971
© 2001 Cancer Research Campaign
doi: 10.1054/ bjoc.2001.1948, available online at http://www.idealibrary.com on IDEAL

http://www.bjcancer.com

### HPV types and cofactors causing cervical cancer in Peru

**C Santos[1], N Muñoz[2], S Klug[3], M Almonte[4], I Guerrero[4], M Alvarez[1], C Velarde[1], O Galdos[1], M Castillo[5], J Walboomers[6], C Meijer[6] and E Caceres[4]**

[1]Instituto de Enfermedades Neoplásicas 'Dr Eduardo Cáceres Graziani', Av. Angamos Este 2520, Lima 34, Peru; [2]International Agency for Research on Cancer, 150, Cours Albert Thomas, F-69372 Lyon cédex 08, France; [3]IARC Fellow, current address: University of Bielefeld, PO Box 100131, D-33501 Bielefeld, Germany; [4]Maes-Heller Cancer Research Centre, Av. Angamos Este 2520, Lima 34, Peru; [5]'María Auxiliadora' General Hospital, Lima, Peru; [6]Free University Hospital, Postbus 7057, NL-1007 MB Amsterdam, The Netherlands

**Summary** We conducted a hospital-based case-control study in Peru of 198 women with histologically confirmed cervical cancer (173 squamous cell carcinomas and 25 cases of adenocarcinoma/adenosquamous carcinoma) and 196 control women. Information on risk factors was obtained by personal interview. Using PCR-based assays on exfoliated cervical cells and biopsy specimens, HPV DNA was detected in 95.3% of women with squamous cell carcinoma and in 92.0% of women with adenocarcinoma/adenosquamous carcinoma compared with 17.7% in control women. The age-adjusted odds ratio was 116.0 (95% CI = 48.6–276.0) for squamous cell carcinoma and 51.4 (95% CI = 11.4–232.0) for adenocarcinoma/adenosquamous carcinoma. The commonest types in women with cervical cancer were HPV 16, 18, 31, 52 and 35. The association with the various HPV types was equally strong for the two most common types (HPV 16 and 18) as for the other less common types. In addition to HPV, long-term use of oral contraceptives and smoking were associated with an increased risk. HPV is the main cause of both squamous cell carcinoma and adenocarcinoma in Peruvian women. © 2001 Cancer Research Campaign http://www.bjcancer.com

**Keywords**: cervical cancer; human papillomavirus; Peru; case-control

The incidence and mortality rates for cervical cancer in Latin America are among the highest in the world. In Peru, the age-standardized incidence rate in 1998 was 40 per 100 000 (IARC, 1998). Cervical cancer is the leading cause of death among Peruvian women, with a mortality rate of 22 per 100 000 (IARC, 1998). It often strikes women during the peak of their economic activity leaving behind young children without mothers. Cervical screening outside the private sector is very limited, covering at most 5% of the poor women (unpublished data from Ministry of Health, Family Planning Programme, 1997). Thus, as in most developing countries, cervical cancer screening programmes in Peru have had little or no impact in reducing the mortality of cervical cancer.

The recognition of a limited number of HPV types as the primary cause of virtually all cervical cancers (Walboomers et al, 1999) has opened new perspectives for the prevention of this malignancy. First, in primary prevention through the use of safe and effective HPV vaccines currently being tested (Coursaget and Muñoz, 1999), and second, in secondary prevention by introducing HPV testing into the screening programmes (Schiffman et al, 2000).

Both strategies require the identification of the relevant HPV types causing cervical cancer in a given population. Towards this aim, the IARC has been conducting a series of case-control studies of cervical cancer in a number of developing countries. We report here the results of an IARC study on invasive cervical cancer in Peru to assess the risk associated with over 30 HPV types and the role of co-factors.

*Received 7 March 2001*
*Revised 1 June 2001*
*Accepted 5 June 2001*

*Correspondence to:* N Muñoz

### MATERIALS AND METHODS

### Study population

Between April 1996 and July 1997, a total of 198 cases and 196 control women were recruited from two hospitals in Lima: Instituto Nacional de Enfermedades Neoplasicas (INEN) and the General Hospital Maria Auxiliadora. Cases were defined as women residing in Lima Metropolitan area with a new histological diagnosis of invasive cervical cancer at the INEN, who have not received any previous treatment, and who were in reasonable physical and mental condition to provide reliable responses to the questionnaire.

Controls were selected from women without cervical cancer or prior history of treatment with conization or hysterectomy attending the same hospital as the cases or the General Hospital. They were selected using broad stratified age matching to the cases within 10-year age groups. Exclusion criteria for the controls included diseases associated with known risk factors for cervical cancer such as cancers of the anogenital tract, tobacco-related cancers and cancer of the breast, endometrium, ovary or colon. The main diagnostic categories of the control subjects included: cholecystitis (31%), inguinal or umbilical hernia (15%), appendicitis (15%), skin diseases (11%), lymphomas and thyroid cancer (11%), pyelonephritis and other infections (11%), benign tumours

(6%), varices (3%), hydatid cysts (2%) and others (3%). The cytological diagnoses of the control women at recruitment were: normal (62%), inflammatory (35%), low-grade squamous intraepithelial lesion (3%). Written informed consent was obtained from all women who participated in the study. The participation rate was 85% for cases and 80% for controls.

### Data and specimen collection

All consenting women were administered a standardized questionnaire by female trained interviewers covering a demographic characteristics, sexual behaviour, reproductive and contraceptive history, genital hygiene, tobacco smoking, coca chewing, screening history and sexually transmitted diseases.

A gynaecological examination was then performed with collection of biopsy specimens in the cases and exfoliated cells in the controls. Necrosis and easy bleeding prevented taking adequate scrapes from most cases. For controls, one Pap smear was prepared and the remaining cells were eluted in phosphate-buffered saline (PBS), pelleted in PBS and stored at –70˚ C for later use for HPV DNA detection. Biopsy specimens from cases were also stored at –70˚ C.

The histological and cytological slides were reviewed by local pathologists.

The study protocol was cleared by the Ethical Committe of the IARC and by a local ethical committee.

### HPV DNA detection and typing

HPV DNA detection and typing was performed using PCR amplification methods at a central laboratory as previously described (de Roda Husman et al, 1994; Jacobs et al, 1995). Briefly, DNA quality was evaluated using β-globin primers and HPV DNA was then amplified using the general primer set GP5+/6+. PCR products were assessed for HPV positivity by low-stringency Southern blot hybridization using a cocktail of HPV specific probes (Walboomers et al, 1992). For viral genotyping the HPV-positive PCR products were successively hybridized with oligonucleotide probes for 33 HPV types (6, 11, 16, 18, 26, 31, 33, 34, 35, 39, 40, 42, 43, 44, 45, 51, 52, 54, 56, 57, 58, 59, 61, 66, 68, 70, 72, 73, IS39, MM4, MM7, CP6108 and CP8304). Viral DNA positive in the GP5+/6+ PCR that did not hybridize with any of the provided probes was arbitrarily named HPV X.

In a second step, all 29 cases positive for β-globin DNA but negative with the GP5+/6+ primers were reamplified with E7 primers for 14 high-risk HPV types (HPV 16, 18, 31, 33, 35, 39, 45, 51, 52, 56, 58, 59, 66 and 68) (Walboomers et al, 1999). A similar number of controls negative with GP5+/6+ primer PCR were randomly selected from those within the same age group as the cases and found positive for the E7 primer system. Of all the GP5+/6+ negative cases ($n$ = 29), 19 were positive with the E7 primer system, while 10 remained negative for HPV. No malignant cells were observed in the tissue sections cut adjacent to those used for HPV DNA detection in these 10 cases. On the other hand, none of the retested controls ($n$ = 18) were HPV DNA-positive with the E7 primer system.

Of the 394 specimens tested, 20 from controls (10.2%) and 2 from cases (1%) were β-globin and HPV-negative (GP5+/6+ primers) and were excluded from statistical analyses. Also, cervical cells from one control (0.5%) were missing, leaving 371 women with HPV data (196 cases and 175 controls).

### Statistical analysis

Odds ratios (ORs) and 95% confidence intervals (Cls) were calculated as approximations of the relative risks by unconditional logistic regression (Breslow & Day, 1980). ORs for the association between histological types of cervical cancer and specific HPV types were adjusted for age categorized in four age-groups (< 40, 40–49, 50–59, > 60). The ORs for the various HPV types were calculated by comparing women who had the specific HPV types (either as a single infection with this type or a multiple infection containing this type) with women who were HPV DNA negative.

To assess the role of risk factors other than HPV, a stratified analysis restricted to HPV DNA positive cases and controls was performed. Statistical significance was tested by the likelihood ratio test for the difference for dichotomous variables and by two-sided linear trend for variables in logically ordered categories.

From variables that were highly correlated (using Pearson's correlation coefficients) and that may have been measuring the same effect, one was selected for inclusion in the final model.

### RESULTS

Out of the 198 cases of invasive cervical cancer, 173 (87.4%) were diagnosed as squamous cell carcinoma, 15 (7.6%) as adenocarcinoma and 10 (5%) as adenosquamous cell carcinomas. According to the FIGO classification, 6 (3%) were categorized as stage I, 101 (51%) as stage II, 85 (43%) as stage III and 6 (3%) as stage IV.

The overall HPV DNA prevalence was 94.9% in cases (95.3% in squamous cell carcinomas and 92% in adenocarcinoma/ adenosquamous carcinomas) and 17.7% among control women (Table 1). However, if we exclude the 10 HPV-negative cases in which no malignant cells were observed in the tissue sections cut adjacent to those used for HPV DNA detection, the prevalence of HPV DNA in cases is 100%.

Fifteen different HPV types were detected among women with squamous cell carcinoma and among control women and five among women with adenocarcinoma/adenosquamous carcinomas. The prevalence of multiple HPV infections among HPV-positive women was 12.9% in squamous cell carcinomas, 8.7% in adenocarcinoma and 22.6% in control women. Table 2 shows that the most common type in women with squamous cell carcinoma was HPV 16 followed by HPV types 18, 31, 52, 33 and 45. In adenosquamous carcinomas HPV 16 was also the most common type followed by HPV 35 and in adenocarcinoma, HPV 16 was the most common type (86%), followed by HPV 18 (21%). In control women, the most common types were HPV 16, 18, 56 and 31.

In 8 (5%) of the women with squamous cell carcinoma and positive for HPV DNA and in 6 (19%) of the control women, the HPV type could not be identified with the 33 type-specific probes that we used and were classified as HPV X. All single HPV infection were caused by high-risk or oncogenic HPV types, except for one low-risk HPV type (11) involving a single control woman. Two low-risk types identified in multiple infections (HPV 6, 42) were found in combination with high-risk types and were also detected in control women only. Among case women the HPV DNA prevalence did not vary with age.

The age-adjusted ORs for the less common HPV types (HPV 33, 35, 39, 52) were as high as those for the most common HPV types (HPV 16 and 18), for both squamous cell carcinomas and adenocarcinomas/adenosquamous carcinomas.

© 2001 Cancer Research Campaign

**Table 1** Type-specific HPV prevalence in cervical cancer patients and control women

| HPV type | Squamous cell carcinoma | | Adenocarcinoma/adenosquamous carcinoma | | Controls | |
|---|---|---|---|---|---|---|
| | ***n*** | **%** | ***n*** | **%** | ***n*** | **%** |
| Negative | 8 | 4.7 | 2 | 8.0 | 144 | 82.3 |
| Positive for any type | 163 | 95.3 | 23 | 92.0 | 31 | 17.7 |
| Total | 171 | 100.0 | 25 | 100.0 | 175 | 100.0 |
| HPV 11 | 0 | 0.0 | 0 | 0.0 | 1 | 3.2 |
| HPV 16 | 89 | 54.9 | 18 | 78.3 | 5 | 16.1 |
| HPV 18 | 10 | 6.2 | 1 | 4.4 | 1 | 3.2 |
| HPV 31 | 14 | 8.6 | 0 | 0.0 | 1 | 3.2 |
| HPV 35 | 1 | 0.6 | 1 | 4.4 | 1 | 3.2 |
| HPV 45 | 2 | 1.2 | 0 | 0.0 | 0 | 0.0 |
| HPV 51 | 1 | 0.6 | 1 | 4.4 | 2 | 6.5 |
| HPV 52 | 10 | 6.2 | 0 | 0.0 | 1 | 3.2 |
| HPV 56 | 1 | 0.6 | 0 | 0.0 | 4 | 12.9 |
| HPV 58 | 2 | 1.2 | 0 | 0.0 | 1 | 3.2 |
| HPV 59 | 1 | 0.62 | 0 | 0.0 | 0 | 0.0 |
| HPV 66 | 2 | 1.2 | 0 | 0.0 | 0 | 0.0 |
| HPV 70 | 0 | 0.0 | 0 | 0.0 | 1 | 3.23 |
| HPV X | 8 | 4.9 | 0 | 0.0 | 6 | 19.4 |
| HPV 6 + 31 | 0 | 0.0 | 0 | 0.0 | 1 | 3.2 |
| HPV 16 + 18 | 0 | 0.0 | 1 | 4.4 | 1 | 3.2 |
| HPV 16 + 31 | 1 | 0.6 | 0 | 0.0 | 0 | 0.0 |
| HPV 16 + 42 | 0 | 0.0 | 0 | 0.0 | 1 | 3.2 |
| HPV 16 + 52 | 1 | 0.6 | 0 | 0.0 | 0 | 0.0 |
| HPV 18 + 31 | 1 | 0.6 | 0 | 0.0 | 0 | 0.0 |
| HPV 18 + 33 | 2 | 1.2 | 0 | 0.0 | 0 | 0.0 |
| HPV 18 + 45 | 3 | 1.9 | 1 | 4.4 | 0 | 0.0 |
| HPV 18 + 52 | 0 | 0.0 | 0 | 0.0 | 1 | 3.2 |
| HPV 18 + 58 | 1 | 0.6 | 0 | 0.0 | 0 | 0.0 |
| HPV 31 + 39 | 1 | 0.6 | 0 | 0.0 | 0 | 0.0 |
| HPV 31 + 52 | 2 | 1.2 | 0 | 0.0 | 0 | 0.0 |
| HPV 31 + 68 | 0 | 0.0 | 0 | 0.0 | 1 | 3.2 |
| HPV 33 + 35 | 2 | 1.2 | 0 | 0.0 | 0 | 0.0 |
| HPV 39 + 45 | 1 | 0.6 | 0 | 0.0 | 0 | 0.0 |
| HPV 52 + 56 | 1 | 0.6 | 0 | 0.0 | 0 | 0.0 |
| HPV 70 +CP8304 | 1 | 0.6 | 0 | 0.0 | 1 | 3.2 |
| HPV 18 + 33 + 35 | 1 | 0.6 | 0 | 0.0 | 0 | 0.0 |
| HPV 18 + 39 + 45 | 1 | 0.6 | 0 | 0.0 | 0 | 0.0 |
| HPV 18 + 33 + 35 + 39 | 1 | 0.6 | 0 | 0.0 | 0 | 0.0 |
| HPV 18 + 33 + 35 + 45 | 1 | 0.6 | 0 | 0.0 | 0 | 0.0 |
| HPV 18 + 33 + 39 + 58 | 1 | 0.6 | 0 | 0.0 | 1 | 3.2 |
| Total multiple types | 22 | 13.5 | 2 | 8.7 | 7 | 22.6 |

Percentage of the single and multiple types were calculated based on the total HPV positive women.

To investigate the relevance of risk factors other than HPV, two types of statistical analyses were performed: a multivariate analysis adjusting for HPV and other potential confounders and a stratified analysis in which only HPV DNA-positive women are included. Only the results of the latter are considered and presented (Table 3). This is because if HPV is the necessary cause of cervical cancer, the role of cofactors should be envisioned only in the progression from HPV carrier state to cancer, when dealing with case-control studies. A significant increase in risk was observed with ever pregnancy, in women who reported using oral contraceptives (OCs) for 4 or more years ($P = 0.005$) and among tobacco smokers and chewers of coca leaves. A significant reduction in risk (OR = 0.2; 95% CI = 0.06–0.6) was observed in those reporting use of intra-uterine device as a contraceptive method. A non-significant increase in risk was observed with lack of schooling, with being a widow, of starting sexual intercourse before 15 years of age and ever practising anal intercourse. Having had more than three lifetime Pap smears and the fact of using sanitary towels were associated with a non-significant reduction in risk. No significant association was detected with the number of sexual partners.

Among control women, the HPV DNA prevalence was lower in women under 40 years of age (10.5%) than among women over 40 (20%), but the difference was not statistically significant ($P = 0.20$). The small number of HPV DNA-positive controls precluded the identification of risk factors for HPV DNA-positivity.

### DISCUSSION

This, the first case-control study of the subject in Peru, revealed that HPV infection is the primary cause of both squamous cell carcinomas and adenocarcinomas/adenosquamous carcinomas in Peru. This is consistent with similar studies carried out by the IARC in Spain and Colombia (Muñoz et al, 1992; Bosch et al, 1993), the Philippines (Ngelangel et al, 1998), Thailand (Chichareon et al, 1998), Brazil (Eluf-Neto et al, 1994), Morocco (Chaouki et al, 1998) and Paraguay (Rolón et al, 2000).

*© 2001 Cancer Research Campaign*

**Table 2** Odds ratios (ORs) for the association between histological types of cervical cancer and HPV types

| | Case subjects | | | | | | | |
|---|---|---|---|---|---|---|---|---|
| | Squamous cell carcinoma | | | Adenocarcinoma/adenosquamous carcinoma | | | Controls | |
| **HPV type (1)** | ***n*** | **%** | **OR* (95% CI)** | ***n*** | **%** | **OR* (95% CI)** | ***n*** | **%** |
| Negative | 8 | 4.7 | 1.0 (referent) | 2 | 8.0 | 1.0 (referent) | 144 | 82.3 |
| Positive for any type | 163 | 95.3 | 102.0 (44.5–236) | 23 | 92.0 | 51.4 (11.4–232) | 31 | 17.7 |
| Total | 171 | 100.0 | 115.9 (48.8–276) | 25 | 100.0 | | 175 | 100.0 |
| HPV 16 | 91 | 55.8 | 255.0 (85.6–759) | 19 | 82.6 | 241 (40.8–1425) | 7 | 22.6 |
| HPV 18 | 22 | 13.5 | 149.0 (33.5–622) | 3 | 13.0 | 40.5 (4.5–364) | 4 | 12.9 |
| HPV 31 | 19 | 11.7 | 238.3 (39.8–1427) | 0 | 0.0 | – | 3 | 9.7 |
| HPV 33 | 8 | 5.0 | 342.8 (26.7–4400) | 0 | 0.0 | – | 1 | 3.2 |
| HPV 35 | 6 | 3.7 | 227.8 (17.7–2926) | 1 | 4.4 | 72.0 (3.2–1604) | 1 | 3.2 |
| HPV 39 | 5 | 3.1 | 176.5 (12.7–2448) | 0 | 0.0 | – | 1 | 3.2 |
| HPV 45 | 8 | 4.9 | ∞ (33.5–∞) | 1 | 4.4 | ∞ | 0 | 0.0 |
| HPV 52 | 14 | 8.6 | 190.3 (28.1–1289) | 0 | 0.0 | – | 2 | 6.5 |
| HPV 58 | 4 | 2.5 | 66.5 (8.0–554) | 0 | 0.0 | – | 2 | 6.5 |
| HPV X | 8 | 4.9 | 37.8 (8.2–174.3) | 0 | 0.0 | – | 6 | 19.4 |
| Other types (2) | 7 | 4.3 | 12.6 (3.5–45.4) | 1 | 4.4 | – | 12 | 38.7 |
| Single infection | 141 | 86.5 | 161.9 (59.4–441) | 21 | 91.3 | 66.6 (14.1–314) | 24 | 77.4 |
| Multiple infection | 21 | 12.9 | 66.6 (14.1–314) | 2 | 8.7 | 19.1 (2.1–175) | 7 | 22.6 |

Percentage of the single and multiple types were calculated based on the total HPV positive women. *Age-adjusted (1) HPV from both single and multiple infection included.(2) includes HPV types 6, 11, 42, 51, 56, 59, 66, 68, 70, CP8304.

HPV DNA ascertainment in cases was done using first L1 primers (GP5+/6+ primers) and subsequently E7 primers in those found HPV-negative with the L1 primers. This retesting increased the HPV DNA prevalence from 85.2% to 94.9%. To assess the possibility that the same could have happened among control women, 18 specimens from control women originally classified as HPV DNA-negative using GP5+/6+ primers and of similar age distribution as the 19 cases retested and found HPV DNA-positive with the E7 primers, were also retested with E7 primers. None of these women were found HPV DNA-positive. This suggests that an underestimation of HPV DNA prevalence in controls leading to an overestimation of the ORs for invasive cervical cancer, is unlikely. The six most common HPV types among cases were: HPV 16 (59.1%), HPV 18 (13.4%), HPV 31 (10.2%), HPV 52 (7.5%), HPV 45 (4.8%) and HPV 33 (4.3%). This type distribution in Peru is similar to that observed in other Latin-American countries (Brazil, Colombia and Paraguay) where HPV types 16, 18 and 31 were the three most common. However, in Peru the fourth and fifth most common types were HPV 52 and 45, while in the other three countries they were HPV 45 and 33. In Latin America, the relative high prevalence of HPV 52 in cervical cancer would appear to be confined to Peru. Among control women, the most common types found (HPV 16, 18) were the same as in women with invasive cervical cancer.

Our results allowed the estimation of the risk of cervical cancer linked to the 9 HPV types most common in this population (HPV 16, 18, 31, 52, 33, 45, 35, 39 and 58). Women in whom two or more different HPV types were detected, did not have a higher risk of cervical cancer than those in whom a single HPV type was detected.

In only 10 cases (5%) HPV DNA could not be detected. It is of interest to note that no malignant cells were observed in the snap-frozen sections of the biopsies that were cut adjacent to those sections used for HPV DNA detection in these 10 cases. Only stroma or necrotic tissue was found in these HPV DNA-negative tissue sections. Thus, if these 10 cases are excluded, the prevalence of HPV DNA in cases would be 100%. These findings are consistent with the results of our IARC international survey based on 1000 cervical cancer biopsy specimens from 22 countries. An initial HPV DNA prevalence of 93% was obtained when L1 primers were used in the initial HPV testing. However, when the specimens originally classified as HPV DNA-negative were retested with E7 primers and strict control of the presence of

© 2001 Cancer Research Campaign

**Table 3** Cofactors for cervical cancer among HPV DNA-positive women

| Variable | % Cases (*n* = 186) | % Controls (*n* = 31) | $OR_1$ (95% CI) | $OR_2$ (95% CI) |
|---|---|---|---|---|
| Pregnancy | | | | |
| Never | 1.6 | 9.7 | 1.0 | 1.0 |
| Ever | 98.4 | 90.3 | 5.8 (1.1–30.8) | 3.5 (0.6–19.7) |
| No. of full-term pregnancies | | | | |
| 0 | 1.6 | 9.7 | 1.0 | 1.0 |
| 1–2 | 15.0 | 9.7 | 7.8 (1.0–59.2) | 5.9 (0.8–45.8) |
| 3–5 | 39.3 | 41.9 | 4.6 (0.8–26.3) | 2.7 (0.5–16.1) |
| ≥6 | 44.1 | 38.7 | 6.5 (1.1–36.9) | 3.6 (0.6–22.0) |
| | | | *P* = 0.02 | *P* = 0.76 |
| Oral contraceptive use | | | | |
| Never | 67.2 | 83.9 | 1.0 | 1.0 |
| Ever | 32.8 | 16.1 | 2.6 (0.9–7.6) | 2.7 (0.9–8.4) |
| Never | 67.2 | 83.0 | 1.0 | |
| ≤3 | 12.9 | 16.1 | 1.0 (0.3–2.9) | |
| ≥4 | 18.8 | 0.0 | ∞ (1.9–∞) | |
| Unknown | 1.1 | 0.0 | | |
| | | | *P* = 0.005 | |
| IUD use | | | | |
| Never | 88.2 | 71.0 | 1.0 | 1.0 |
| Ever | 11.8 | 29.0 | 0.3 (0.1–0.7) | 0.2 (0.06–0.6) |
| Smoking | | | | |
| Never | 90.3 | 100.0 | 1.0 | |
| Ever | 9.7 | 0.0 | ∞ (0.8–∞) | |
| | | | *P* = 0.08 | |
| Coca chewing | | | | |
| Never | 93.0 | 100.0 | 1.0 | |
| Ever | 7.0 | 0.0 | ∞ (0.6–∞) | |
| | | | *P* = 0.22 | |

$OR_1$ = Odds ratios adjusted for age; $OR_2$ = Odds ratios adjusted for age, screening history, age at first intercourse, ever pregnancy, ever use of oral contraceptives.

malignant cells was done, the prevalence increased to 99.7% (Walboomers et al, 1999).

Our results indicate that HPV is the primary and probably a necessary cause of cervical cancer in Peru. However, it is not a sufficient cause. To assess the role of cofactors in this and in our previous studies, we have used two analytical strategies. In the first, we included all women independently of their HPV DNA status, and accounted by the strong effect of HPV by statistical adjustment. However, this type of adjustment is inappropriate if we consider that a small proportion of cases are most probably false negative, and the vast majority of controls are not carriers of HPV and therefore not at risk of developing cervical cancer. Thus, we have presented here only the results obtained with the second strategy, that is, restricting the analysis to HPV DNA-positive cases and controls. However, because of the small number of HPV DNA-positive controls, our ability to detect significant associations is rather limited. Despite the above, our analyses restricted to HPV DNA-positive women allowed the identification of long-term use (> 5 years) of OCs as the principal cofactor in this population.

Long-term use of OCs was also identified as a cofactor in our studies in Spain and Colombia (Bosch et al, 1994); Brazil (Eluf-Neto et al, 1994), the Philippines (Ngelangel et al, 1998), Morocco (Chaouki et al, 1998) and Paraguay (Rolon et al, 2000). However, the association of OC use and CIN-3 appears to be less consistent; while some studies have reported an increased risk with long-term use (Muñoz et al, 1993; Lacey et al, 1999), others have not found a clear association (Deacon et al, 2000). The mechanism by which OCs increase the risk of cervical cancer in carriers of HPV DNA has not been elucidated. It has been suggested that steroid hormones may facilitate the integration of HPV DNA (Mittal et al, 1993).

There was also a suggestion that pregnancy, smoking and chewing coca leaves may be potential cofactors. The epidemiological evidence linking smoking with cervical cancer is controversial (Szarewski and Cuzick, 1998). Analyses of HPV DNA-positive cases and controls have shown an association with smoking in some of our previous studies (Chichareon et al, 1998; Ngelangel et al, 1998), but not in others (Bosch et al, 1992; Eluf-Neto et al, 1994). A clear increased risk and a significant dose-response relationship for CIN-3 have been recently reported (Deacon et al, 2000). The association with the habit of chewing coca leaves is intriguing, but it is based on small numbers.

In summary, our study has identified the HPV types causing cervical cancer in Peru and also long-term use of oral OCs, smoking and coca chewing as potential cofactors. Lack of power due to the small number of HPV DNA-positive controls in each study precluded definitive conclusions. A proper evaluation of these cofactors is currently underway in a pooled analysis of all our case-control studies.

### ACKNOWLEDGEMENTS

The study was funded by the International Agency for Research on Cancer. The authors acknowledge the contribution of Mrs A. Arslan in the data management and statistical analysis, the

 *© 2001 Cancer Research Campaign*

collaboration of the following Peruvian physicians: J. Mariategui, C. Castellano, O. Barriga, R. Galdos, from the Instituto de Enfermedades Neoplósicas and C. Perez and J. Jeroónimo from 'Maria Auxiliadora' General Hospital and of the social worker Gabriela Leonardo.

### REFERENCES

Bosch FX, Muñoz N, de Sanjosé S, Navarro C, Moreo P, Ascunce N et al (1993) Human papillomavirus and cervical intraepithelial neoplasia grade III/carcinoma in situ: a case-control study in Spain and Colombia. *Cancer Epidemiol, Biom Prev* **2**: 415–422

Bosch FX, Muñoz N, Sanjosé S, Eluf-Neto J, Orfila J, Walboomers J and Shah K (1994) What is relevant in cervical carcinogenesis other than HPV? In: Papillomavirus in Human Pathology, Monsonego J (ed). *Challenges of Modern Medicine* **9**: 173–181

Breslow NE and Day NE (1980) *Statistical methods in cancer research. Vol. 1: The analysis of case-control studies (IARC Scientific Publications No 32)*. International Agency for Research on Cancer: Lyon

Chaouki N, Bosch FX, Muñoz N, Meijer CJLM, EI Gueddari B, El Ghazi A et al (1998) The viral origin of cervical cancer in Rabat, Morocco. *Int J Cancer* **75**: 546–555

Chichareon S, Herrero R, Muñoz N, Walboomers JMM, Jacobs M, Bosch FX et al (1998) Risk factors for cervical cancer in Thailand: a case-control study. *J Natl Cancer Inst* **90**: 50–57

Coursaget P and Muñoz N (1999) Vaccination against infectious agents associated with human cancer. *Cancer Surveys* **33**: 355–381

Deacon JM, Evans CD, Yule R, Desai M, Binns W, Taylor C and Peto J (2000) Sexual behaviour and smoking as determinants of cervical HPV infection and of CIN3 among those infected: a case-control study nested within the Manchester cohort. *Br J Cancer* **88**: 1565–1572

de Roda Husman AM, Walboomers JMM, Meijer CJLM, Risse EKJ, Schipper MEI, Helmerhorst TJM et al (1994) Analysis of cytomorphologically abnormal cervical scrapes for the presence of 27 mucosotropic human papillomavirus genotypes using polymerase chain reaction. *Int J Cancer* **56**: 802–806

Eluf-Neto J, Booth M, Muñoz N, Bosch FX, Meijer CJLM and Walboomers JMM (1994) Human papillomavirus and invasive cervical cancer in Brazil. *Br J Cancer* **69**: 114–119

Family Planning Programme (1997) National Plan for Gynaecological Cancer Prevention, unpublished report, Ministry of Health: Lima, Peru

Higgins DG and Sharp PH (1988) Clustal: a package for performing sequence alignment on a microcomputer. *Gene* **73**: 237–244

IARC (1998) *Globocan I, Cancer Incidence and Mortality Worldwide (IARC Cancer Base No 3)*. International Agency for Research on Cancer: Lyon

Jacobs MV, de Roda Husman AM, van den Brule AJC, Snijders PJF, Meijer CJLM and Walboomers JMM (1995) Group-specific differentiation between high- and low-risk human papillomavirus genotypes by general primer-mediated PCR and two cocktails of oligonucleotide probes. *J Clin Microbiol* **33**: 901–905

Lacey JV Jr, Brinton LA, Abbas FM, Barnes WA, Gravitt PE, Greenberg MD et al (1999) Oral contraceptives as risk factors for cervical adenocarcinomas and squamous cell carcinomas. *Cancer Epidemiol Biomarkers Prev* **8**: 1079–1085

Mittal R, Pater A and Pater MM (1993) Multiple human papillomavirus type 16 glucocorticoid response elements functional for transformation, transient expression, and DNA-protein interactions. *J Virol* **67**: 5656–5659

Muñoz N, Bosch FX, de Sanjosé S, Tafur L, Izarzugaza I, Gili M et al (1992) The causal link between human papillomavirus and invasive cervical cancer: a population-based case-control study in Colombia and Spain. *Int J Cancer* **52**: 743–749

Muñoz N, Bosch FX, de Sanjosé S, Vergara A, del Moral A, Muñoz MT, Tafur L et al (1993) Risk factors for cervical intraepithelial neoplasia grade III/carcinoma in situ in Spain and Colombia. *Cancer Epidemiol Biomark Prev* **2**: 423–431

Ngelangel C, Muñoz N, Bosch FX, Limson GM, Festin MR, Deacon J et al (1998) The causes of cervical cancer in the Philippines: a case-control study. *J Natl Cancer Inst* **90**: 43–49

Rolón PA, Smith JS, Muñoz N, Klug SJ, Herrero R, Bosch FX et al (2000) Human papillomavirus infection and invasive cervical cancer in Paraguay. *Int J Cancer* **85**: 486–491

Schiffman M, Herrero R, Hildesheim A, Sherman M, Bratti M, Wacholder S et al (2000) HPV DNA testing in cervical cancer screening. Results from women in a high-risk province of Costa Rica. *J Am Med Assoc* **283**: 87–93

Szarewski A and Cuzick J (1998) Smoking and cervical neoplasia: a review of the evidence. *J Epidemiol Biostat* **3**: 229–256

Walboomers JMM, Jacobs MV, Manos MM, Bosch FX, Kummer JA, Shah KV et al (1999) Human papillomavirus, a necessary cause of invasive cervical cancer worldwide. *J Pathol* **189**: 12–19

© 2001 Cancer Research Campaign
